# Ecosystem Scale Acoustic Sensing Reveals Humpback Whale Behavior Synchronous with Herring Spawning Processes and Re-Evaluation Finds No Effect of Sonar on Humpback Song Occurrence in the Gulf of Maine in Fall 2006

**DOI:** 10.1371/journal.pone.0104733

**Published:** 2014-10-07

**Authors:** Zheng Gong, Ankita D. Jain, Duong Tran, Dong Hoon Yi, Fan Wu, Alexander Zorn, Purnima Ratilal, Nicholas C. Makris

**Affiliations:** 1 Department of Electrical and Computer Engineering, Northeastern University, Boston, Massachusetts, United States of America; 2 Department of Mechanical Engineering, Massachusetts Institute of Technology, Cambridge, Massachusetts, United States of America; Pacific Northwest National Laboratory, United States of America

## Abstract

We show that humpback-whale vocalization behavior is synchronous with peak annual Atlantic herring spawning processes in the Gulf of Maine. With a passive, wide-aperture, densely-sampled, coherent hydrophone array towed north of Georges Bank in a Fall 2006 Ocean Acoustic Waveguide Remote Sensing (OAWRS) experiment, vocalizing whales could be instantaneously detected and localized over most of the Gulf of Maine ecosystem in a roughly 400-km diameter area by introducing array gain, of 18 dB, orders of magnitude higher than previously available in acoustic whale sensing. With humpback-whale vocalizations consistently recorded at roughly 2000/day, we show that vocalizing humpbacks (i) were overwhelmingly distributed along the northern flank of Georges Bank, coinciding with the peak spawning time and location of Atlantic herring, and (ii) their overall vocalization behavior was strongly diurnal, synchronous with the formation of large nocturnal herring shoals, with a call rate roughly ten-times higher at night than during the day. Humpback-whale vocalizations were comprised of (1) highly diurnal non-song calls, suited to hunting and feeding behavior, and (2) songs, which had constant occurrence rate over a diurnal cycle, invariant to diurnal herring shoaling. Before and during OAWRS survey transmissions: (a) no vocalizing whales were found at Stellwagen Bank, which had negligible herring populations, and (b) a constant humpback-whale song occurrence rate indicates the transmissions had no effect on humpback song. These measurements contradict the conclusions of Risch et al. Our analysis indicates that (a) the song occurrence variation reported in Risch et al. is consistent with natural causes other than sonar, (b) the reducing change in song reported in Risch et al. occurred days before the sonar survey began, and (c) the Risch et al. method lacks the statistical significance to draw the conclusions of Risch et al. because it has a 98–100% false-positive rate and lacks any true-positive confirmation.

## Introduction

Passive acoustic survey methods employing hydrophones at fixed locations [1]–[15] or mobile platforms [16], [17] have been widely used to detect, localize, track and study the behavior [1]–[9], [13]–[15] and abundance [4], [10]–[12] of whales. With our array situated on the northern flank of Georges Bank from September 19 to October 6, 2006 [18], [19], we could detect and localize vocalizing whales over most of the Gulf of Maine, a roughly 400-km diameter area, including Georges and Stellwagen Banks, and so monitor vocalization behavior over an ecosystem scale. This was possible because we used a large-aperture, densely-sampled, coherent hydrophone array with orders of magnitude higher array gain [20]–[25] than previously available in acoustic whale sensing. We detected roughly 2000 humpback whale vocalizations per day and used these to determine the corresponding whale locations over time by introducing a synthetic aperture tracking technique [26]–[29] and the array invariant method [30] to the whale sensing problem.

We find that the distribution of the vast majority of vocalizing humpback whales coincided with the primary time and location of Atlantic herring during their peak annual spawning period. During daylight hours, herring were found to be dispersed on the seafloor in deeper waters over wide areas of Georges Bank's northern flank [18]. At sunset, they would then rise and converge to form dense and massive evening shoals, which migrated to the shallow waters of Georges Bank for spawning, following a regular diurnal pattern [18]. We find the humpback whale vocalization behavior followed a similarly strong diurnal pattern, temporally and spatially synchronous with the herring shoal formation process, with vocalization rates roughly ten times higher at night than during daylight hours. At night, most humpback whale vocalizations originated from concentrated regions with dense evening herring shoals, while during daytime, their origins were more widely distributed over areas with significant but diffuse pre-shoal herring populations. These vocalizations are comprised of: (i) non-song calls, dominated by repetitive downsweep “meows” (approximately 1.44 second duration, 452 Hz center frequency, 170 Hz bandwidth, and 31 second repetition rate) which apparently have not been previously observed; and (ii) songs [2]. The repetitive non-song calls were highly diurnal and synchronous with the herring shoal formation process, consistent with hunting and feeding behavior. In contrast, songs occurred at a constant rate with no diurnal variation, and are apparently unrelated to feeding and the highly diurnal herring spawning activities.

Before and during Ocean Acoustic Waveguide Remote Sensing (OAWRS) survey transmissions [18], [19], we measured constant humpback whale song occurrence, indicating these transmissions had no effect on humpback whale song. In addition, our data shows no humpback whale vocal activity originating from Stellwagen Bank, which had negligible herring populations [31], [32], but vocalizing humpbacks located near Georges Bank, which had dense and decadally high herring populations [31], could be heard at Stellwagen Bank. These results are consistent with previous observations of humpback whale feeding activity in the Gulf of Maine and Stellwagen Bank which show humpback whales leave Stellwagen Bank for other regions plentiful in herring for feeding during the herring spawning season [33]. These results, however, contradict the conclusions of Risch et al. [34]. To investigate this contradiction, the Risch et al. statistical test [34] is applied to the annual humpback whale song occurrence time series reported from single sensor detections at Stellwagen Bank in time dependent ambient noise published by Vu et al. [35] and shown to false-positively find that humpback whales react to sonar 98–100% of the time over a yearly period when no sonars are present. A simple explanation for this severe statistical bias [36], [37] is found upon inspection of the Vu et al. [35] multi-annual humpback whale song occurrence time series. The reported time series [35] have (i) inconsistencies in trend, (ii) large differences in song occurrence, and (iii) random correlation between years when no sonar is present. This shows that 98–100% of the time, the approach used in Risch et al. [34] mistakes natural variations in song occurrence for changes caused by sonar when no sonar is present. When the Risch et al. statistical test [34] is applied to the same humpback whale song occurrence data reported in Risch et al. [34] for 2008 and 2009, it false-positively finds humpback whales respond to sonar 100% of the time when no sonar is present. With the 98–100% false positive rate and the lack of any true positive confirmation for the Risch et al. statistical approach [34], the analysis of Risch et al. [34] lacks the statistical significance to draw the conclusions found in Risch et al. [34]. The fact that the reported reducing change in humpback whale song occurrence, to zero [34], [35], occurred while the OAWRS vessels were docked on the other side of Cape Cod from Stellwagen Bank, at the Woods Hole Oceanographic Institution, due to severe winds, days before OAWRS transmissions for active surveying began on September 26, 2006, yet no other explanation for this reduction than sonar is provided in Risch et al. [34], is consistent with a violation of temporal causality in the Risch et al. [34] study. Our data analysis indicates that the change in humpback whale song occurrence Risch et al. [34] reported is consistent with wind-dependent noise [20], [23], [38], [39] limiting the single-hydrophone measurements of Risch et al. [34] to a small wind-speed-dependent fraction of the singing humpback whales and songs detected by our densely sampled, large aperture, coherent array. These findings are all consistent with the constant humpback whale song occurrence rates before and during OAWRS survey transmissions found with our wide-area towed array measurements.

## Results and Discussion

### 2.1 Humpback whale behavior is synchronous with herring spawning processes during the peak annual Atlantic herring spawning period in the Gulf of Maine

**Figure 1:**
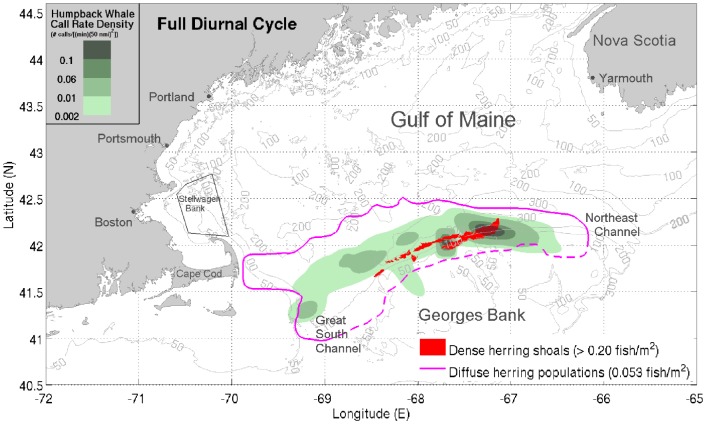
Distributions of vocalizing humpback whales and spawning herring populations in Fall 2006. Spatial distribution of vocalizing humpback whales coincides with the time and location of spawning Atlantic herring distributions in Fall 2006. Humpback whale vocalizations are found to be distributed along the northern flank of Georges Bank, coinciding with dense herring shoals (>0.20 fish/m^2^, red shaded areas) imaged using active OAWRS system [18] and diffuse herring populations (≈0.053 fish/m^2^, bounded by magenta line) obtained from conventional fish finding sonar (CFFS) line-transect data from NEFSC Annual Fall Herring Surveys [18], [63]. The green shaded areas indicate the overall humpback whale call rate densities (number of calls/[(min) (50 nmi)^2^]) measured with our large aperture array. All data represent means between September 22 and October 6, 2006. The dashed magenta line represents the southern bound of the NEFSC survey tracks [18], [63]. The black trapezoid indicates Stellwagen Bank [158].

**Figure 2:**
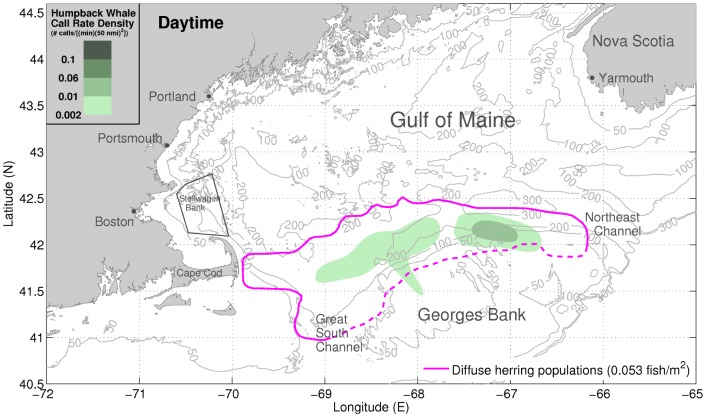
Daytime distributions of vocalizing humpback whales and diffuse herring populations. Spatial distribution of vocalizing humpback whales coincides with the locations of diffuse herring populations during daytime hours. In daylight, the vast majority of the humpback whale vocalizations originate within areas containing diffuse herring populations (≈0.053 fish/m^2^, bounded by magenta line) [63]. The green shaded areas indicate the daytime humpback whale call rate densities (number of calls/[(min) (50 nmi)^2^]) measured with our large aperture array. All data represent daytime means between September 22 and October 6, 2006. The dashed magenta line represents the southern bound of the NEFSC survey tracks [18], [63]. The daytime hours are between sunrise and sunset (06:00:01 to 18:00:00 EDT). The black trapezoid indicates Stellwagen Bank [158].

**Figure 3:**
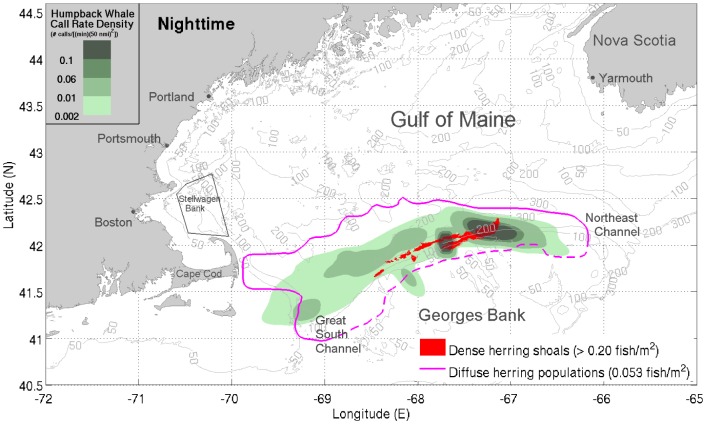
Nighttime distributions of vocalizing humpback whales and dense herring shoals. Spatial distribution of vocalizing humpback whales coincides with the locations of dense evening herring shoals during nighttime hours. At night, vocalizing humpback whales become concentrated at and near dense evening herring shoals (>0.20 fish/m^2^, red shaded areas) that form along the northern flank of Georges Bank and call rates increase dramatically [18]. The green shaded areas indicate the nighttime humpback whale call rate densities (number of calls/[(min) (50 nmi)^2^]) measured with our large aperture array. All data represent nighttime means between September 22 and October 6, 2006. The magenta line bounds the areas with diffused herring populations (≈0.053 fish/m^2^). The dashed magenta line represents the southern bound of the NEFSC survey tracks [18], [63]. The data shown are for nighttime hours between sunset and sunrise the next day (18:00:01 to 06:00:00 EDT). The black trapezoid indicates Stellwagen Bank [158].

Vocalizing humpback whales and spawning herring populations [18], [19] were simultaneously localized and imaged over thousands of square kilometers during the peak annual spawning period of Atlantic herring in the Gulf of Maine by instantaneous passive and active OAWRS [18], [40], [41] techniques respectively in the Fall of 2006. We find humpback whale behavior in the Gulf of Maine to be highly coupled to peak herring spawning activities, which last for roughly one week but whose inception can vary [42], [43] by many weeks from year to year. This coupled humpback whale and herring behavior occurs over too short a period to be accurately resolved by available seasonal, yearly or decadal averages [44], [45], but can be well resolved by OAWRS methods. The high array gain [20]–[25] of the densely sampled large aperture coherent OAWRS passive receiver array used here enables detection of whale vocalizations either two orders of magnitude more distant in range or lower in signal-to-noise ratio (SNR) than a single hydrophone (Sections 3.1 and 3.5), which has no array gain. The array used here has 160 hydrophones with 4 nested 64-hydrophone subapertures. We determined whale bearings by beamforming and ranges by applying the instantaneous array invariant method [30] and synthetic aperture tracking techniques [26]–[29], [46] to the whale sensing problem, leading to the spatial distribution of humpback whale call rate density shown in Figures 1, 2 and 3 over the period from September 22 to October 6, 2006, which coincided exactly with the peak annual herring spawning period [42]. Humpbacks are identified based on presence of song, as well as appropriate frequency content, duration, signature and repetition rate of calls.

We find that the vast majority of vocalizing humpback whales were spatially distributed in regions coinciding with the primary aggregations of spawning herring during the peak annual herring spawning period [18], [42] in the Gulf of Maine (Figure 1). During this period, spawning herring populations instantaneously imaged by the active OAWRS system were found to regularly form massive dense shoals during evening hours along the northern flank of Georges Bank between water depths of 50 m and 200 m, which constituted the favorable shoal formation areas [18], [19] (Figure 1). Water depths of 160 to 200 m were favored by spawning herring to form dense and massive evening shoals (>0.20 fish/m^2^), before migration to shallower water (≈50 m) spawning grounds on Georges Bank [18]. The more diffusely scattered herring populations with lower areal population density (≈0.053 fish/m^2^) were found to be widely distributed between water depths of 50 m and 300 m, which include dense shoal formation areas [18], by concurrent Northeast Fisheries Science Center (NEFSC) line-transect ultrasound and trawl surveys [47], as shown in Figures 1, 2, and 3. At night, most vocalizing humpback whales were also found to be concentrated within water depths of 50 m to 300 m, in close proximity to the dense evening herring shoals (Figure 3). During daytime, vocalizing humpbacks were widely distributed within regions containing the more diffuse pre-shoal herring populations on the northern flank of Georges Bank and the Great South Channel (Figure 2). The observed high spatial correlation between the distribution of vocalizing humpback whales and the primary spawning herring populations in the Gulf of Maine is consistent with a mass feeding of humpback whales on herring that is synchronized with the peak herring spawning processes.

**Figure 4:**
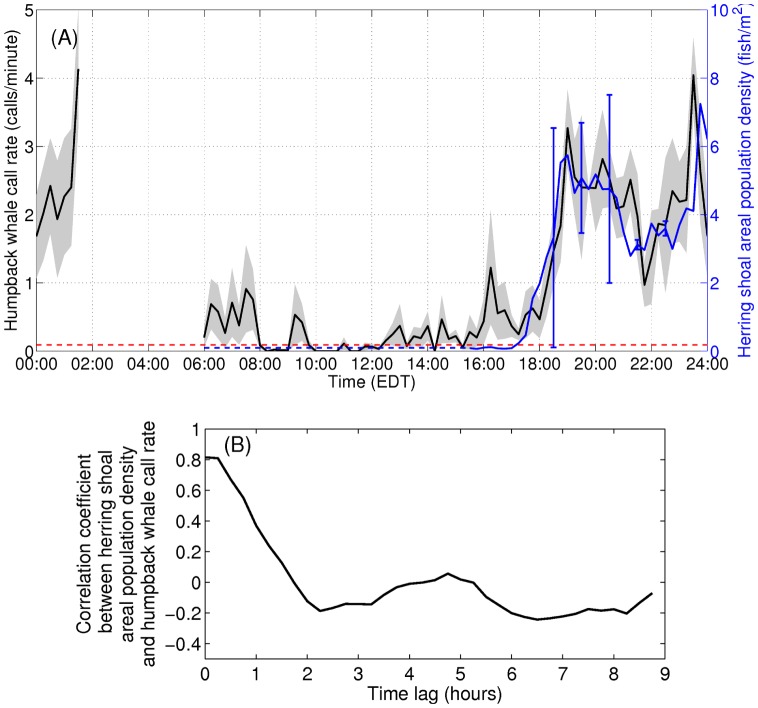
Humpback whale call-rate is synchronized with Atlantic herring shoal population density over a diurnal cycle. (A) Mean humpback whale call rate (black line within gray standard deviation over 15 minute bins) over a diurnal cycle and mean herring shoal areal population density (blue line with standard deviation indicated by the blue error bars) from September 28 to October 3. When the areal population density of the diffuse daytime herring populations reaches a critical threshold of approximately 0.2 fish/m^2^ (red dashed line) near sunset, the herring population density drastically increases at a rate of roughly 5 fish/m^2^ per hour [18] to form evening shoals. (B) Diurnal humpback whale call rate follows a synchronous pattern with 0.82 correlation coefficient and 0–15 minute time lag between the two time series in (A). The period from roughly 2–6 EDT contains a data gap.

We find humpback whale vocalization behavior follows a strong diurnal pattern that is temporally synchronous with the regular herring shoal formation process [18]. The diurnal pattern is quantified by vocalization rates roughly ten times higher at night than during daylight hours (Figure 4(A)). The synchronization is quantified by a high correlation (0.82 at 0–15 minute time lag in Figure 4(B)) between time series of spawning herring shoal population density and humpback whale call rate (Figure 4(A)).

The mechanisms behind the observed synchronized diurnal pattern between humpback whales and spawning herring can be understood by examining the shoal formation process. In daytime, the herring are more widely distributed within thin layers roughly 5 m from the seafloor (on average 0.053 fish/m^2^) in deeper waters on the northern flank of Georges Bank (Figure 2A of Ref. [18]). Near sunset local convergences of population density reach a critical threshold of 0.2 fish/m^2^ after which coherent shoal formation waves appear (Figures 1 to 3 of Ref. [18]) and areal population density drastically increases at a rate of roughly 5 fish/m^2^ per hour (Figure 3 of Ref. [18]) to form dense and massive shoals. Shoal formation in deeper waters after dusk allows herring spawning activities to proceed under the cover of darkness with reduced risk of predator attack [48], [49]. The resulting roughly 50-fold increase in the areal population density of herring shoals, triggered by reduction in light levels, is closely followed (within 15 minutes) by a sudden order of magnitude increase in humpback whale call rate, as shown in Figure 4. The corresponding spatial focusing of vocalizing humpback whales from regions containing the overall dispersed herring populations in the day to those with dense shoals at night has been shown in Figures 2 to 3. Evening humpback whale vocalization rates remain high during the subsequent migration of herring shoals toward shallower spawning grounds on Georges Bank [18], and throughout the night until herring shoals dissipate as light levels increase at sunrise [18] (Figure 4). These findings are consistent with a feeding-behavior cause for the elevated humpback whale nocturnal vocalization rates and spatial focusing on dense shoals. The findings of vocal humpback whales exclusively in the vicinity of large spawning herring aggregates during the peak annual herring spawning period, and diurnal vocalization rates synchronized with diurnal herring spawning processes, also provide substantial evidence in favor of the theory that humpback whales leave areas with negligible herring populations, and migrate to primary herring spawning grounds in the Gulf of Maine where large herring populations make hunting and feeding far more efficient [33].

**Figure 5:**
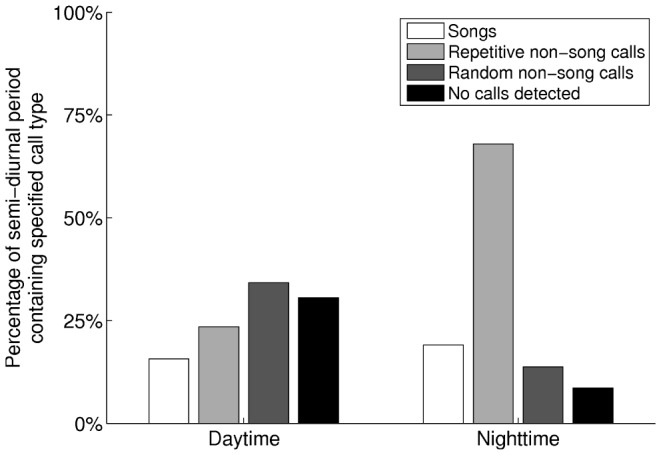
Percentage of semi-diurnal period containing different classes of humpback whale vocalizations for day and night. A roughly three-fold percentage increase is found at night for repetitive non-song calls, which are primarily responsible for the overall diurnal dependence of observed humpback whale vocalizations. Humpback whale songs showed negligible mean variation compared to standard deviations for day (15.7%±18%) versus night (19.1%±15%). Percentages were calculated using the approaches discussed in Section 3.2. The total percentage, the sum of all four categories, exceeds 100% because different call types could occur within overlapping time windows. The “No calls detected”, however, is mutually exclusive with the other categories. Here the daytime hours are between sunrise and sunset (06:00:01 to 18:00:00 EDT) and nighttime hours are between sunset and sunrise the next day (18:00:01 to 06:00:00 EDT).

**Figure 6:**
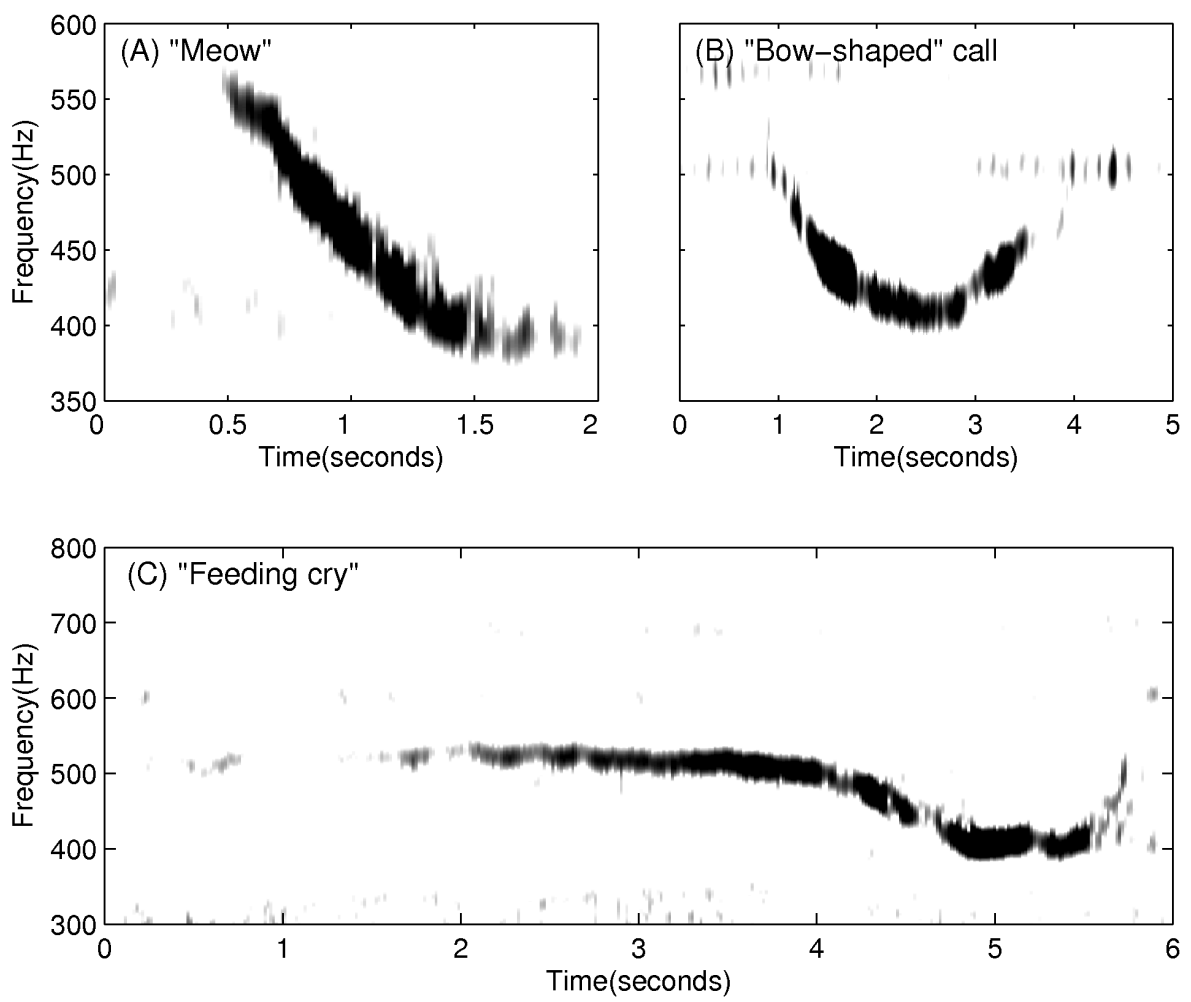
Spectrograms of a typical “meow”, “bow-shaped” call and “feeding cry” observed during OAWRS 2006 experiment. (A) “Meow” is a roughly 1.4 second duration, frequency modulated downsweep signal (570 to 380 Hz) with a center frequency of roughly 475 Hz. (B) “Bow-shaped” call has a roughly 2.4 second duration, downsweep frequency modulated section (510 to 395 Hz) followed by a short upsweep coda with a center frequency of roughly 440 Hz. (C) “Feeding cry” consists of (1) a main section that lasts approximately 3.5 seconds with frequency oscillations between 500 Hz and 540 Hz and (2) a 2 second long frequency-modulated ending section.

**Figure 7 pone-0104733-g007:**
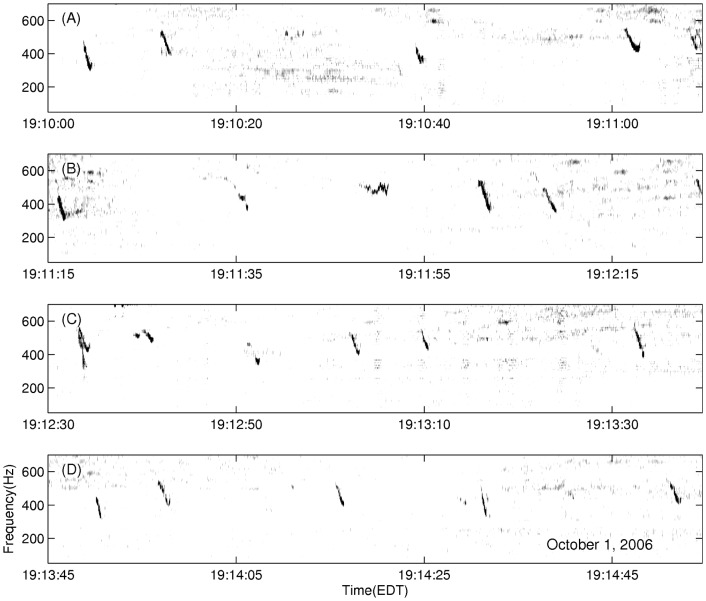
Spectrograms of typical repetitive “meows” observed during OAWRS 2006 experiment in the Gulf of Maine. Four 70-s time series containing repetitive meows are shown in (A) – (D) recorded 5-s apart, on October 1, 2006 between 19:10:00 EDT and 19:14:55 EDT.

The diurnal nature of observed humpback whale vocalizations (Figure 4) is comprised of a three-fold occurrence rate increase of repetitive non-song calls at night (Figure 5), which is consistent with communication [50]–[52] or prey echolocation [50], [51], [53] during feeding activities. “Meows” are the most frequently recorded non-song calls at night, followed by “bow-shaped” calls and “feeding cries”. Repetitive “meows” are primarily uttered in series at night, in spatial and temporal synchronization with the formation of large spawning herring shoals. They are characterized by roughly 1.44 second duration, frequency modulated (537 Hz to 367 Hz) downsweep signals repeated at roughly 31 second intervals (Figures 6(A) and 7). Apparently, they have not been previously observed. These “meows” have significantly different spectral-temporal structure from “Megapclicks” [54], which are of much higher frequency, higher repetition rate, and lower source level, and have been previously associated with evening foraging activities. It has been suggested in Ref. [54] that “Megapclicks” could be “useful for some form of rough acoustic detection such as identifying the seafloor or other large target.” Apart from communication, another possible function of “meows” could be to detect large targets, in particular large prey aggregations. Moreover, the range resolution for acoustic sensing using the finite time duration “meow” calls is *cT*/2≈1 km [18], [21], [26], [27], [40], [55]–[62], without matched filter pulse compression, where *c* is the sound speed and *T* is the time duration of the “meows,” and so is consistent with echolocation of large herring shoals that typically exceed 1 km in horizontal extent [18], [19], [63]–[65]. Previously observed humpback whale “cries” [66] of roughly 0.4–8.2 second duration occur in a frequency band overlapping with that of “meows,” but are characterized by shorter, frequency modulated introductory and ending sections, separated by a relatively longer middle section with less frequency modulation, making them significantly different from the observed “meows.” Individually uttered “meows”, which only occurred intermittently with no pattern, were observed over the full diurnal cycle, and were far less numerous than repetitive “meows” uttered in series. The “bow-shaped” calls are the second most abundant humpback whale non-song vocalizations observed at night. Similar to the repetitive “meows,” they are also primarily uttered in series at night. The “bow-shaped” calls are characterized by a repetition interval of roughly 58 seconds, a roughly 2.36 second duration, a frequency modulated (511 to 367 Hz) main downsweep section followed by a short upsweep coda (Figure 6(B)), and a repetition interval roughly 2 times longer than that of the repetitive “meows”. The humpback whale “feeding cries” we observed are characterized by a roughly 3.18 second duration, frequency oscillating main pulse followed by a short highly frequency modulated coda (Figure 6(C)). They occurred only at night but far less frequently than the repetitive “meows” and “bow-shaped” calls, with a repetition interval of roughly 11 minutes. The “feeding cries” we observed are similar in frequency band and duration to individual “cries” previously observed in Alaskan humpback whale cooperative feeding [66], which is consistent with the calls we observed being related to cooperative humpback whale feeding on spawning herring.

**Figure 8:**
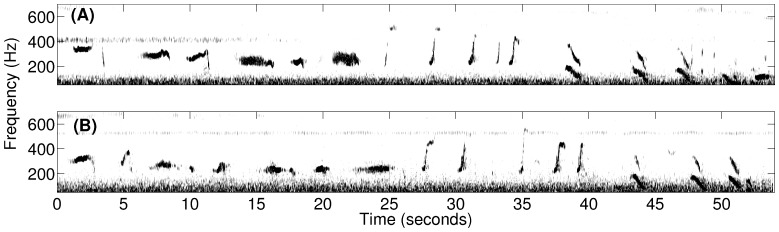
Spectrograms of a typical repeated humpback whale song theme observed during OAWRS 2006 experiment. A repeated humpback whale song theme, starting at (A) 23:17:44 EDT and (B) 23:49:01 EDT and each lasting roughly 1 minute, was recorded on October 2, 2006 from a singing humpback whale in the northern flank of Georges Bank.

Humpback whale songs ([Fig pone-0104733-g007] Figure 8) were found to lack diurnal variation across our observations during the peak annual herring spawning period (Figure 5), which is consistent with an invariance of singing behavior to diurnal feeding activities. Months before the herring spawning season and far from prime herring spawning grounds, absence of diurnal variation was previously observed in humpback whales singing north of the Great South Channel, which was thought to be potentially related to aseasonal mating [67]. In contrast, a diurnal pattern in acoustic energy was detected off of Western Maui, Hawaii, during the humpback whale breeding season with a single omni-directional hydrophone [5]. The increased acoustic energy at night was in the humpback whale vocalization band and attributed to humpback whale song choruses in breeding activities. The fact that songs occurred far less frequently than non-song calls in our observations by a factor of 4 (Figure 5), is consistent with humpback whale vocalization behavior that is closely related to primary seasonal activities.

### 2.2 Re-evaluation finds no effect of sonar on humpback whale song occurrence

**Figure 9:**
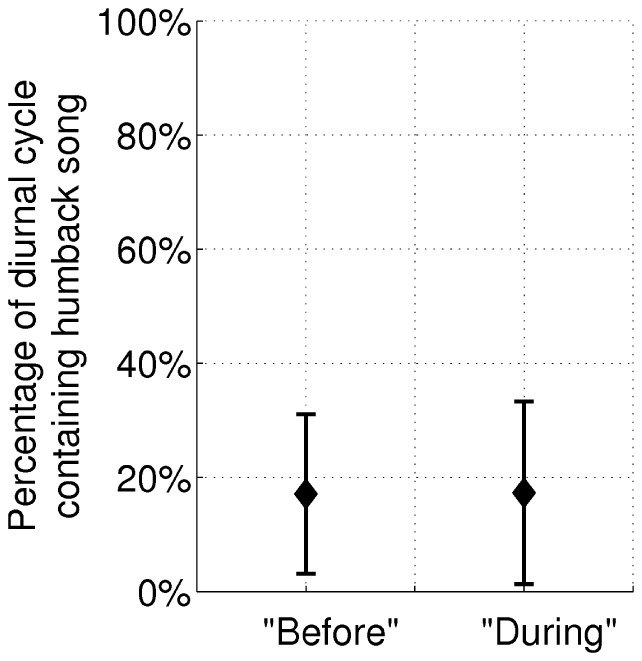
Humpback song occurrence rate is constant in the periods “before” and “during” OAWRS survey transmissions. The mean percentage of a diurnal cycle containing humpback whale song in the periods “before” and “during” OAWRS survey transmissions, as defined in Section 2.2, remains constant, indicating the transmissions had no effect on humpback whale song over the entire passive 400-km diameter survey area of the Gulf of Maine including Stellwagen Bank.

Before and during OAWRS survey transmissions [18], [19], we measured a constant humpback whale song occurrence rate, as shown in Figure 9, indicating no change of humpback song related to these transmissions over the entire survey area in the Gulf of Maine, a roughly 400-km diameter area, including Georges and Stellwagen Banks. Additionally, we find that the humpback whale song occurrence rate from Stellwagen Bank was constant before and during OAWRS survey transmissions, indicating no change of humpback song at Stellwagen Bank related to these transmissions. These direct measurements contradict the conclusions of Risch et al. [34].

To investigate this contradiction, we first follow the standard practice of checking for the bias [36], [37] of a statistical test by applying the test to control data where no stimulus is present to determine the false positive outcome rate [68]–[70]. Since the bias of Risch et al. statistical test [34] was not checked in Risch et al. [34], we do so here (Section 3.4) with the available annual humpback whale song occurrence data [35] from the same set of single sensors Risch et al. [34] used at Stellwagen Bank. We show that their statistical test false-positively finds whales react to sonar 98–100% of the time over a yearly period when no sonars are present. For example, when their statistical test is applied to annual humpback whale song occurrence data published in Ref. [35], with 2006 as the test year and 2008 as the control year, it false-positively finds whales react to sonar: (1) 100% of the time over the year before the “during” period; and (2) 98% of the time over the year when the “during” period is excluded from the test, as described in Section 3.4 and Table 1. Here the “during” period is defined as the 11-day period from September 26 to October 6 with active OAWRS survey transmissions, the “before” period is the 11-day period before the “during” period, and the “after” period is the 11-day period after the “during” period following the usage in Risch et al. [34]. When applied to the same humpback whale song occurrence data reported in Risch et al. [34] over the 33-day period from September 15 to October 17 for 2008 and 2009, with either of these two years as the test year and the other as the control year, the statistical test false-positively finds humpback whales respond to sonar 100% of the time when no sonar is present, as described in Section 3.4 and Table 2, indicating a self-contradiction in the Risch et al. [34] approach. No meaningful conclusions can be drawn from a statistical test with such high bias.

**Table 1:** Percentage of time the Risch et al. statistical test [34] incorrectly finds whales respond to sonar when no sonar is present using annual humpback whale song occurrence data reported from single sensor detections at Stellwagen Bank [35] in time-dependent ambient noise.

Analysis period	Excluding “during” period^a^	Before “during” period^a^
% of time with false-positive response	98.0%(49/50)	100%(35/35)

Risch et al. statistical test [34] is applied to all continuous 33-day periods, as described in Section 3.4.1, in the annual humpback whale song occurrence reported from single sensor detections at Stellwagen Bank in 2006 and 2008 [35], with 2006 as the test year and 2008 as the control year. The test false-positively finds humpback whales react to sonar 98–100% of the time over a yearly period when no sonars are present. The fraction of time when the Risch et al. statistical test [34] false-positively finds whales react to sonar is given in the parenthesis. The parenthetical numbers in the denominator represent the total number of 33-day periods with no sonar present within the analysis period and the parenthetical numbers in the numerator represent the number of 33-day periods when the Risch et al. statistical test [34] false-positively finds whales react to sonar when no sonar is present.

a The “during” period is defined in Section 2.2.

**Table 2:** The Risch et al. statistical test is applied to the same humpback whale song occurrence data reported in Risch et al. [34] over the 33-day period from September 15 to October 17 for 2008 and 2009, with either of these two years as the test year and the other as the control year.

Risch et al. statistical test	Result
With 2008 as the test year and 2009 as the control year	**False positive response**
With 2009 as the test year and 2008 as the control year	**False positive response**

It false-positively finds that whales react to sonar 100% of the time when no sonar is present, indicating self-contradictions in the Risch et al. [34] approach.

**Figure 10:**
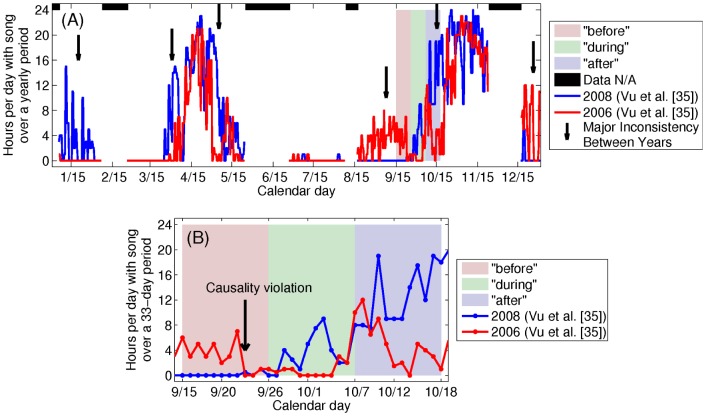
Reported humpback whale Stellwagen Bank song occurrence [35] shows large natural variations within and across years. Large natural variations in humpback whale song occurrence reported from single sensor detections at Stellwagen Bank [35] in time-dependent ambient noise within and across years are common in the absence of sonar. Line plots of reported single sensor daily humpback whale song occurrence at Stellwagen Bank in hours/day (A) for the entire year and (B) from September 15 to October 17, in 2006 and 2008 [35]. Many periods lasting roughly weeks where high song occurrence episodes are found in one year but not in another when no sonars are present are indicated by black arrows in (A). The reported reducing change in humpback whale song occurrence, to zero [34], [35], occurred in the “before” period while the OAWRS vessels were inactive and docked on the other side of Cape Cod from Stellwagen Bank, at the Woods Hole Oceanographic Institution, due to severe winds for days before OAWRS transmissions for active surveying began on September 26, 2006, as marked by the black arrow in (B). This shows that Risch et al. [34] analysis violates temporal causality.

**Figure 11:**
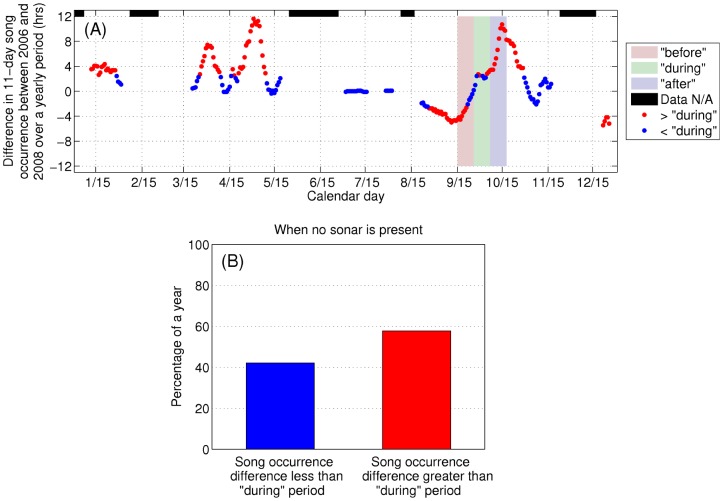
Quantifying large differences in the reported humpback whale song occurrence at Stellwagen Bank [35] across years. Difference in humpback whale song occurrence reported from single sensor detections at Stellwagen Bank [35] in time-dependent ambient noise across years exceeds that of the “during” period most of the time when no sonars are present. (A) Difference in mean humpback whale song occurrence at Stellwagen Bank over respective 11-day periods with 1-day increment in 2006 and 2008, (B) histogram of difference in mean humpback song occurrence over 11-day periods between 2006 and 2008 when no sonar is present, i.e. excluding the “during” period from September 26 to October 6. Periods when the difference in means of respective 11-day periods is greater than (red dots) and less than (blue dots) that of the “during” period are indicated in (A). The difference in means fluctuates randomly throughout the year, exceeding the “during” period 57.8% of the time (most of the time) when no sonars are present, indicating that there is nothing unusual about such differences, which are actually the norm.

An explanation for the severe bias in the statistical test of Risch et al. [34] becomes evident upon inspection of the annual humpback whale song occurrence time series published in Ref. [35]. Very large natural variations within and across years are common in the humpback whale song occurrence time series when no sonars are present, as can be seen in Figure 10. There are many periods lasting roughly weeks where high song occurrence episodes are found in one year but not in another, when no sonars are present (Figure 10). For the majority of the time, greater than 57%, the difference in the song occurrence across years when no sonars are present exceeds that of the “during” period (Figure 11), indicating that there is nothing unusual about such differences, which rather than “alterations” [34] are actually the norm. The statistical test used by Risch et al. [34] is overwhelmingly biased because it mistakes natural variations in humpback whale song occurrence 98–100% of the time for changes caused by sonar when no sonar is present, lacks any true positive confirmation and so lacks the statistical significance to draw the conclusions of Risch et al. [34].

**Figure 12:**
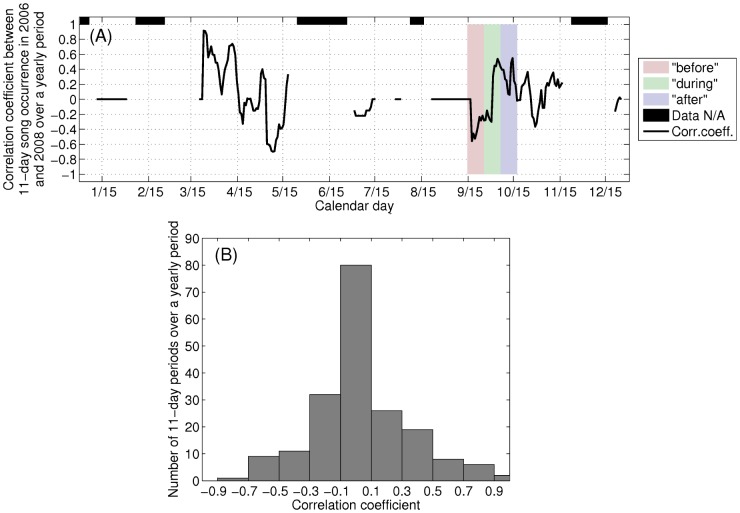
Reported annual humpback song occurrence at Stellwagen Bank [35] are uncorrelated between years over 11-day periods. Annual humpback whale song occurrence reported from single sensor detections at Stellwagen Bank [35] in time-dependent ambient noise are uncorrelated over 11-day periods across years. (A) Correlation coefficient between 2006 and 2008 humpback whale song occurrence time series over 11-day period with 1-day increment (B) histogram of the correlation coefficient in (A). The correlation coefficient of the annual humpback whale song occurrence time series over 11-day periods across years obeys a random distribution peaking at zero correlation about which it is symmetric, showing that correlation in trend between years is random and quantitatively expected to be zero with roughly as many negative correlations as positive ones. The correlation coefficient between the humpback whale song occurrence across years smoothly transitions from negative values in the “before” period, showing no similarity or relation in trend between years just before the 2006 OAWRS survey transmission period, to some of the highest positive correlations obtained between years in the “during” period. This demonstrates high similarity and relation in trend between years during the 2006 OAWRS active survey transmission period, which contradicts the results of the Risch et al. [34] study.

Since the reported reducing change in humpback whale song occurrence, to zero [34], [35], occurred in the “before” period (Figure 10) while the OAWRS vessels were inactive and docked on the other side of Cape Cod from Stellwagen Bank at the Woods Hole Oceanographic Institution due to severe winds days before OAWRS transmissions for active surveying began on September 26, 2006, the Risch et al. analysis [34] severely violates temporal causality. Moreover, the annual humpback whale song occurrence time series are uncorrelated over 11-day periods across years, and the correlation coefficient obeys a random distribution peaking at zero correlation about which it is symmetric (Figure 12), showing that correlation in trend between years is random and quantitatively expected to be zero with roughly as many negative correlations as positive ones. In fact, the correlation coefficient between the humpback whale song occurrence across years smoothly transitions from negative values in the “before” period, showing no similarity or relation in trend between years just before the 2006 OAWRS survey transmission period, to some of the highest positive correlations obtained between years in the “during” period (Figure 12). This demonstrates high similarity and relation in trend between years during the 2006 OAWRS active survey transmission period, which contradicts the results of the Risch et al. [34] study. These causality violations are also discussed in the context of the measured temporal coherence of humpback whale song occurrence in Section 3.6.

**Figure 13:**
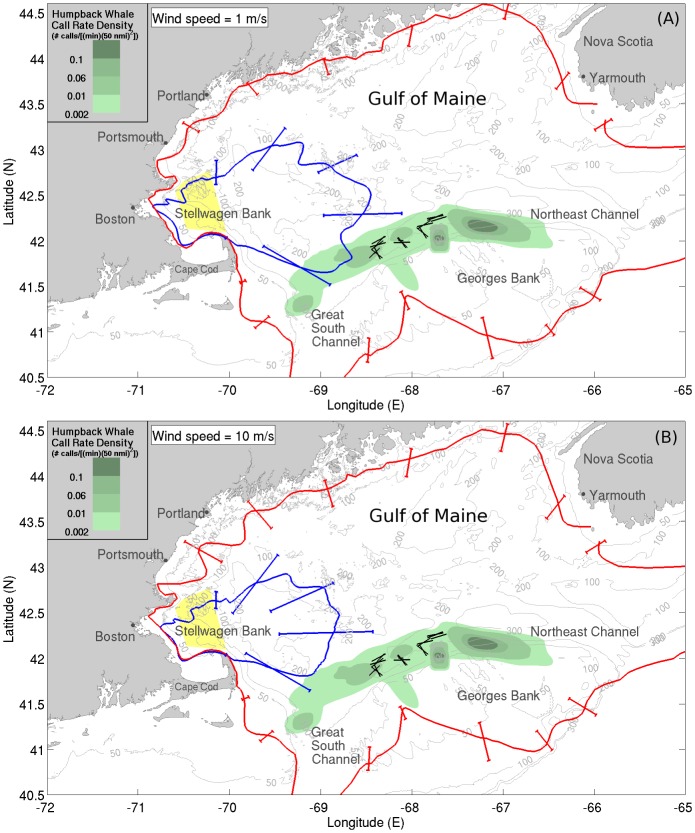
Wind-dependence of mean detection range for single sensor at Stellwagen Bank[34], and OAWRS receiver array. The green shaded areas indicate the overall vocalizing humpback whale call rate densities (number of calls/[(min) (50 nmi)^2^]) determined between September 22 and October 6, 2006 by our large aperture receiver array towed along several tracks (black lines). The mean detection ranges for the single sensor at Stellwagen Bank are in blue and for the OAWRS receiver array are in red, where Stellwagen Bank is marked by yellow shaded regions. These detection ranges are determined by the methods described in Section 3.5 given a humpback whale song unit source level of approximately 180 dB re 1 *µ*Pa and 1 m which is the median of all published humpback whale song source levels [93], [101], [102], [152]–[154]. The error bars represent the spread in detection range due to typical humpback whale song source level variations (Section 3.5). Under (A) low wind speed conditions vocalizing whales are within the mean detection area for a single Stellwagen Bank sensor but for (B) higher wind speeds most vocalizing whales are outside the mean detection area of the same sensor, which results in reduction of detectable whale song occurrence by the single sensor [34] at Stellwagen Bank.

**Figure 14:**
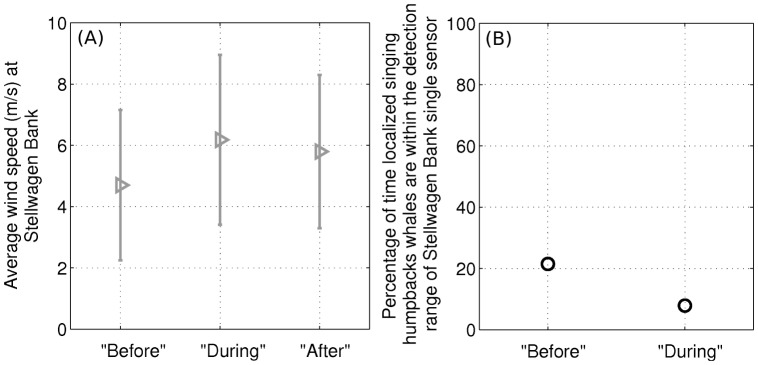
Wind-speed increase causes reduction in humpback song occurrence at Stellwagen Bank. Average wind speed increase from the “before” to the “during” period at Stellwagen Bank causes reduction in the percentage of time humpback whale songs are within mean detection range of a single Stellwagen Bank sensor. (A) Averaged wind speed measured at the NDBC buoy [72] closest to Stellwagen Bank over the “before,” “during,” and “after” 11-day periods; and (B) percentage of the time vocalizing humpback whales localized by our large aperture array are within the mean detection range of the single sensor [34] at Stellwagen Bank in the “before” and “during” periods, using waveguide propagation methods and whale song parameters described in Section 3.5. Since the OAWRS experiment was conducted only up to October 6, 2006, the humpback whale source distribution in the “after” period was not measured and we do not investigate the percentage of time that humpback whales are within the mean detection range of the single sensor at Stellwagen Bank [34] for the “after” period. The triangles represent the mean wind speed and the solid ticks represent the standard deviation of the wind speed over the respective 11-day periods.

**Figure 15:**
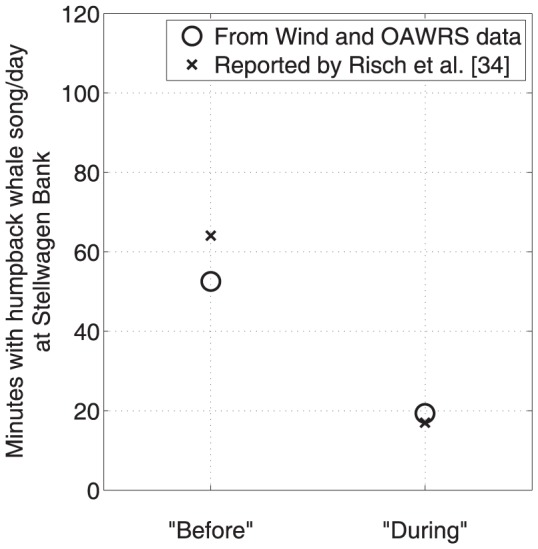
Humpback song occurrence detectable by single sensor matches reported humpback song occurrence at Stellwagen Bank [34]. Average humpback whale song occurrence detectable by a single hydrophone at Stellwagen Bank in time-dependent ambient noise in the “before” and the “during” periods matches the reported humpback whale song occurrence by Risch et al. [34]. Using the measured wind speeds at Stellwagen Bank [72] (Figure 14), the measured spatial distribution of vocalizing humpback whales (Figure 1), and constant song production rates (Figure 9) measured by our large-aperture array, the detectable song occurrence over the “before” and “during” period are found to be within ±18% of the reported means [34], much less than the standard deviations of reported song occurrence[34], using waveguide propagation methods and whale song parameters described in Section 3.5. Before and during OAWRS survey transmissions, this figure shows that reported variations in song occurrence at Stellwagen Bank by Risch et al. [34] are actually due to detection range changes caused by wind-dependent ambient noise, through well established physical processes [20], [73].

It is well known that wind speed variation can lead to severe detection range limitations in passive sensors, especially a single sensor that has zero array gain [20], [23], [25], [71]. Risch et al. [34] did not investigate the effect of wind dependent ambient noise on the detection range of their single hydrophones located in the Stellwagen Bank (Figure 13). They did report that “Ambient noise levels over the whole analysis bandwidth (10–1000 Hz) and in the frequency band with most humpback whale song energy (70–300 Hz) did not vary dramatically within or between years.” Wind speeds varied, however, from calm to near-gale conditions within a period of a few hours or days, many times over the 33-day period examined by Risch et al. [34], as is common for Fall in Stellwagen Bank [72]. These natural wind speed variations must have significantly changed the local wind-dependent noise level according to known physics [20], [73]. Since noise “can have a tremendous, if not a dominating, influence on the detection range of any sonar system” [39], the dramatic changes in wind speed at Stellwagen Bank must have led to dramatic changes in the detection range of single sensors deployed there. The range at which signals, in this case humpback whale songs, can no longer be detected because they become indistinguishable from ambient noise is the detection range from the sensor. Since ambient noise is wind speed dependent, so is the detection range (Figure 13), and so is humpback whale song occurrence measured at that sensor if variations in wind speed cause the detection range to pass through the range of the singing humpback whales (Figure 14). In this case even if a whale sang at a constant rate, song occurrence measured at the sensor (Figure 15) would vary with local wind noise (Figure 14). Moreover, the annual humpback whale song occurrence reported in Ref. [35] had a standard deviation of 3.54 dB in the 33-day period examined by Risch et al. [34], which was less than the 3.8 dB standard deviation in ambient noise level reported by Risch et al. [34], and so local ambient noise variation could have caused all the variations in humpback whale song occurrence reported over that period.

Using the measured wind speeds at Stellwagen Bank [72], and the measured spatial distribution and constant rates of singing humpback whales determined by our large aperture array, we determine the song occurrence detectable by a single hydrophone at Stellwagen Bank, as shown in Figure 15. We find it to match the song occurrence reported by Risch et al. [34] in the “before” and “during” periods with high accuracy, within ±18% of the reported means, which is much less than the standard deviation of the humpback whale song occurrence reported by Risch et al. [34]. This match shows that the variation in reported song occurrence from the “before” to “during” period is due to detection range limitations of the single sensor at Stellwagen Bank from wind-dependent ambient noise, and is not due to the song production rate, which we show to be constant. The constant song production and occurrence rates in the “before” and “during” periods measured by our large aperture array are unaffected by wind noise because the array gain was sufficiently high to make the detection range well beyond the range of the vocalizing whales for all wind conditions (Figure 13). Our data shows no humpback whale vocal activity originating from Stellwagen Bank in either the “before” or “during” periods, but vocalizing humpback whales located near Georges Bank could be heard at Stellwagen Bank during low wind noise conditions (Figure 13). In high wind noise, the single sensor mean detection range at Stellwagen Bank is too short to include the regions with measured singing humpback whales, but in low wind noise, it is large enough to include the regions with measured singing humpback whales as shown in Figure 13, making the mean song detection rate at Stellwagen Bank higher in lower wind noise. Noise from near gale force winds in the last 3 days of the “before” period, for example, caused a significant drop in the detection range of the single sensor and the corresponding significant drop in the song occurrence rate at Stellwagen Bank [35] while the OAWRS vessels were inactive and docked at the Woods Hole Oceanographic Institution. Since the OAWRS experiment was conducted only up to October 6, 2006, the vocalizing humpback whale distribution in the “after” period was not measured and we do not investigate the song occurrence for that period.

It has been previously shown that due to collapse of the herring stock at Stellwagen Bank, humpback whale populations drastically decline at Stellwagen Bank during the herring spawning period and correspondingly increase at other locations where spawning populations are large [33]. Moreover, in the Fall of 2006, herring populations were negligible in the Massachusetts Bay and Cape Cod area, including Stellwagen Bank [32], but in contrast were decadally high in the Georges Bank region [31], consistent with the theory that humpback whales migrate to locations with large spawning herring aggregations [33]. This phenomenon was not mentioned or investigated in Risch et al. [34], but it is highly relevant because the time period Risch et al. [34] focused on is centered exactly on the peak annual herring spawning period of the Gulf of Maine for 2006. Indeed, it has been previously shown by OAWRS in Ref. [18] and by annual NEFSC acoustic echosounding and trawl surveys in Refs. [63] and [43] that this peak annual herring spawning period occurred from the last week of September to the first week of October 2006 on Georges Bank. Based on the results of Ref. [33], it should then be expected that the Stellwagen Bank humpback whale population would be low at this time and the population at Georges Bank would be high, as has been confirmed in Section 2.1 for vocalizing humpback whales.

The levels of the various anthropogenic noises at Stellwagen Bank were not discussed in Risch et al. [34], but only OAWRS levels were selected for analysis and discussion without this context. It is recommended by the National Academy of Sciences (NAS), however, that “A comprehensive noise impact assessment would include additional specific data regarding both sound levels and sources throughout the area for which impacts are being assessed [74].” Such an impact assessment should include “all aspects of the acoustic environment” [75] to avoid the problem another impact assessment had of being evaluated as “misrepresentative of the existing soundscape [74].” Here the soundscape of anthropogenic noise sources at Stellwagen Bank, from highest to lowest intensity or loudest to most quiet is delineated in Tables 3 and 4, following these NAS recommendations, where it is seen that the reported OAWRS transmissions fell at the quietest end of the noise spectrum when audible. Shipping traffic, on the other hand, contributes most to the anthropogenic component of mean acoustic intensity at Stellwagen Bank by many orders of magnitude. Most anthropogenic sources of underwater noise listed in Tables 3 and 4 continuously operate [76], [77] over a wide range of frequencies audible to whales, i.e. tens to hundreds of Hertz [20], [39], [77], [78], and result in received levels that may exceed the currently recommended NOAA guideline of 120 dB re 1 µPa received level [79]–[83] in water for continuous noise [84] for a range of whale distances (Table 3). Even the maximum OAWRS received sound pressure level reported by Risch et al. [34] is orders of magnitude lower than the current 160 dB NOAA guideline for short duration signals such as the OAWRS 1–2 seconds duration pulse, and significantly lower than the 120 dB guideline for even continuous sources [84] which OAWRS is not. The maximum received acoustic intensities of OAWRS signals at Stellwagen Bank reported by Risch et al. [34] are the same as those of a quiet wooded forest or a quiet room with no conversation [85], whereas the acoustic intensities received at Stellwagen Bank from shipping traffic are often the same as those of a busy roadway or a busy airport runway [26], [85]. Risch et al. [34] reported that visual inspections of humpback whales in Stellwagen Bank were made during the OAWRS experiment, suggesting that humpback whales were within visible range of research vessels. Research vessels close enough to whales to sight them can easily have engine noise levels at the whales greatly exceeding the reported OAWRS levels over broader frequency bands and much greater time duration (Table 3).

**Table 3:** Typical anthropogenic noise sources at Stellwagen Bank.

Continuous anthropogenic noise source	Source level in dB re 1 *µ*Pa and 1 m	Frequency in Hz	Source range in km for received level above 120^a^ dB re 1 *µ*Pa	Source range in km for received level between 88–110^b^ dB re 1 *µ*Pa	Acoustic intensity in Watts/m^2^ 1 m away from anthropogenic noise source
Cruise ship	219 [159]	10 to >1,000 [160]	<100	160 to >200	5,000
Cargo vessel	192 [20], [161]	10 to >1,000 [20], [161]	<10	30–200	10
Research vessel	166–195 [159]	40 to >1,000 [77], [159]	<6	2–130	0.025–20
Outboard motor boat	176 [78], [162]	100 to >1,000 [163], [164]	<2	3–20	0.25
Whale watching boat	169 [165]	100 to >1,000 [165]	<1	3–25	0.05

a Recommended received pressure level in the NOAA guideline for continuous-type sources [84].

b Range of received pressure level at Stellwagen Bank single sensor reported by Risch et al. of OAWRS impulsive signal [34], of roughly 1–2 seconds duration and at least 75 seconds spacing between impulses. Source ranges are determined at the frequencies with maximum humpback whale vocalization energy, using the waveguide propagation methods described in Section 3.5. Humpback whale vocalizations are known to have source levels in the range of 175 to 188 dB re 1 *µ*Pa and 1 m [9], [101], [102], [153], and have been reported to go up to 203 dB re 1 *µ*Pa and 1 m [166]. All data shown in the table is for sources and measurements in water where 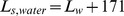 based on the sound speed and density of water, 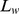 is the power level in dB re 1 Watt, and 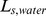 is the source level in dB re 1 *µ*Pa and 1 m. Underwater noise from a typical low flying jet airplane [26] can lead to underwater sound pressure levels exceeding 120 dB re 1 *µ*Pa in water at ranges less than 5 kilometers.

**Table 4:** Received mean intensity of typical anthropogenic noise sources at Stellwagen Bank.

Continuous anthropogenic noise source	Received level in water in dB re 1 *µ*Pa (or corresponding mean intensity in Watts/m^2^) 500 m a away from an anthropogenic noise source over a minute or longer	How many decibels higher (or times greater) the mean intensity of the given anthropogenic noise source over a minute or longer at 500 m is than that reported for OAWRS at Stellwagen Bank [34]
Cruise ship	177 (0.33)	85 (300,000,000)
Cargo vessel	147 (0.00033)	55 (300,000)
Research vessel	121–144 (0.00000083–0.00017)	29–52 (750–150,000)
Outboard motor boat	131 (0.0000083)	39 (7,500)
Whale watching boat	124 (0.0000017)	32 (1,500)

a Whale watching vessels [167] are allowed to approach humpback whales at ranges much less than 500 m according to NOAA Whalewatching Guidelines [168].

Before and during OAWRS survey transmissions, we measured constant humpback whale song occurrence and production rates over our entire survey area roughly 400-km in diameter covering most of the Gulf of Maine, including Stellwagen Bank, indicating the transmissions had no effect on humpback whale song production rate. Using annual humpback whale song occurrence reported from single sensor detections at Stellwagen Bank [35] in time dependent ambient noise, we show the statistical test used by Risch et al. [34] for assessing the response of humpback whales to sonar transmission false positively finds humpback whales respond to sonar 98–100% of the time when no sonars are present. With this and the lack of any true positive confirmation for the Risch et al. [34] statistical approach, the analysis of Risch et al. [34] lacks the statistical significance to draw the conclusions of Risch et al. [34]. The fact that the Risch et al. [34] analysis only allows sonar causes for the reducing change reported in Risch et al. [34], yet the change occurred days before the sonar survey began, is consistent with a violation of temporal causality in the Risch et al. [34] study. The Risch et al. statistical test [34] mistakes natural variations in whale song reception, from such factors as natural variations in whale distributions [44], singing behavior [1], [2], and ambient noise, for changes caused by sonar 98–100% of the time when no sonar is present. Before and during OAWRS survey transmissions, we find that the variations in song occurrence at Stellwagen Bank reported by Risch et al. [34] are consistent with the natural phenomena of detection range fluctuations caused by wind-dependent ambient noise, through well established physical processes [20], [73]. Misinterpretation of natural phenomenon from flawed analytic methods such as biased testing and neglect of physical laws can have seriously negative consequences [86]–[90].

## Materials and Methods

### 3.1 The passive receiver array

Acoustic recordings of whale vocalizations were acquired using a horizontal passive receiver line-array, the ONR five-octave research array [91], towed by Research Vessel *Oceanus* along designated tracks just north of Georges Bank [18], [19], as shown in Figure 13. The multiple nested sub-apertures of the array contain a total of 160 hydrophones spanning a frequency range from below 50 to 3750 Hz for spatially unaliased sensing. A fixed sampling frequency of 8000 Hz [19] was used so that acoustic signals with frequency contents up to 4000 Hz were recorded without temporal aliasing. Two linear apertures of the array, the low-frequency (LF) aperture and the mid-frequency (MF) aperture, both of which consist of 64 equally spaced hydrophones with respective inter element spacing of 1.5 m and 0.75 m, were used to analyze humpback whale calls with fundamental frequency content below 1000 Hz. For humpback whale calls with frequency content below 500 Hz, the LF aperture was used, while for humpback whale calls with frequency content extending beyond 500 Hz up to 1 kHz, the MF aperture was used. The angular resolution 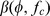 of the horizontal receiver array is 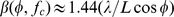 for broadside (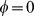) through angles near endfire (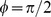), where 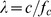 is the acoustic wavelength, *c* is the sound speed, *f_c_* is the center frequency, and *L* is the array aperture length. At endfire, the angular resolution is 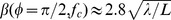. Permission for this National Oceanographic Partnership Program experiment was given in the Office of Naval Research document 5090 Ser 321RF/096/06.

### 3.2 Measurement and analysis of humpback whale vocalizations

Acoustic pressure time series measured by sensors across the receiver array were converted to two-dimensional (2D) beam-time series by time-domain beamforming [20], [22], [25], [26], and further converted to spectrograms by temporal Fourier transform. Whale vocalizations were detected and characterized in time and frequency for each azimuth by visual inspection.

**Figure 16:**
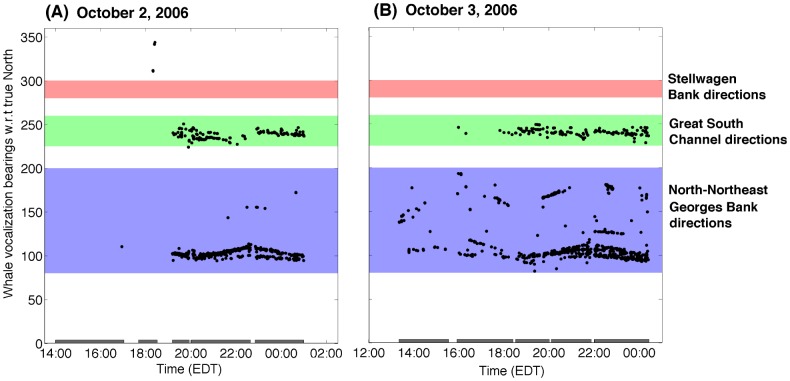
Vocalizing humpback whale bearings measured by our large-aperture receiver array. Examples of vocalizing humpback whale bearings measured on (A) October 2 and (B) October 3, 2006. Almost all humpback whale vocalizations are found to originate from North-Northeast Georges Bank directions (purple shaded areas) and the Great South Channel directions (green shaded areas), but none originates from Stellwagen Bank directions (red shaded areas). All vocalizing humpback whale bearings are measured from the true North in clockwise direction with respect to the instantaneous spatial locations of towed horizontal receiver array center. The techniques used here for resolving source bearing ambiguity about the horizontal line-array's axis are described in Section 3.3. The shaded bars on the *x*-axis indicate the operation time periods of the towed array.

With our densely sampled, large-aperture array, multiple vocalizing humpback whale individuals could be tracked in beam-time and compared with the bearings of historic humpback whale habitats in the Gulf of Maine, including the Georges Bank, Stellwagen Bank, Great South Channel, and Northeast Channel as shown in Figure 16. Throughout our entire experiment, including the “before” and “during” periods discussed in Section 2.2, we measured roughly 2000 humpback whale vocalizations per day but none originated from Stellwagen Bank, as in the Figure 16 example.

As noted in Section 2.1, both humpback whale song [1], [2], [8], [67], [92]–[94] and non-song [6], [7], [9], [54], [66], [95] vocalizations were measured, where non-song vocalizations contained repetitive and random calls. Songs [2] were composed of repeating themes, which could be sub-divided into phrases and units. A song session typically consisted of at least two themes and often lasted over tens of minutes, with gaps of silence not exceeding ten minutes between any two themes. An example of repeated song themes is shown in Figure 8. Repetitive non-song calls were defined as series of downsweep “meows” or “bow-shaped” calls, which contained at least two similarly structured “meows” or “bow-shaped” calls that were uttered within a short time interval of roughly 31 seconds or 58 seconds, respectively. Random non-song calls, were primarily composed of individual “meows”, “bow-shaped” calls, and “feeding cries” that occurred at least one minute apart from any type of individually uttered non-song calls. We found that roughly 73% of the non-song vocalizations were “meows,” roughly 22% were “bow-shaped” calls, and roughly 5% were “feeding cries.” These non-song calls were observed in the frequency range of 250–700 Hz (Table 5). The standard and primary method of using spectral and temporal characteristics of the vocalizations to identify whale species [6], [34], [35], [95]–[100] is used here. The specific spectral and temporal characteristics of calls we observed are provided in Table 5, following a standard approach for classifying calls established by Dunlop et al. [6]. Since all non-song calls or non-song call sequences we detected *consistently* originated or ended at the the same spatial position as song calls, to within our reported position error in Section 3.3, and occurred immediately after or before these co-located song calls, alternating with song calls, it is most likely that the same species and group of whales produced the song and non-song calls we report. Given this and the fact that humpback whales are the only species known to produce song in this region, season and frequency range, it is most likely that the non-song calls we report are also from humpback whales and extremely unlikely that they originate from other species. Furthermore, humpback whales are the most abundant, by 1–2 orders of magnitude, vocalizing whales in the 250–700 Hz frequency range [2], [6], [7], [9], [101], [102] in the Gulf of Maine during the fall season [45]. While North Atlantic right whales, minke whales and sei whales have been observed to rarely vocalize solely in the 250–700 Hz frequency range, it is also unlikely that the non-song calls we observed were produced by these whales because (1) right and minke whale tonal calls are roughly 4–8 times shorter in time duration or roughly a factor of 2 lower in frequency than the non-song calls we observed [103]–[107]; (2) the typical right whale “gunshot” calls are of a much broader frequency content than 250–700 Hz and are more than an order of magnitude shorter in time duration than the non-song calls we observed [103], [104], [106], [108]; (3) the more typical minke whale “pulse trains” lasting tens of seconds are comprised of pulses that are more than an order of magnitude shorter in time duration and have a minimum frequency roughly a factor of 2 lower than that of the non-song calls we observed [109], [110]; (4) right whales are 20 times less abundant, minke whales are 10 times less abundant, and sei whales are 60 times less abundant than humpback whales in the Gulf of Maine during the fall season [45]; (5) sei whales have not been observed to vocalize in the 250–700 Hz frequency range in the North Atlantic and the North Pacific [111]–[114]; and (6) previous work shows humpback whales to be by far the dominant consumers of herring on Georges Bank of the whales that have been observed to vocalize in the 250–700 Hz range, where right and sei whales appear to consume negligible amounts of herring [115]. There were numerous sightings of humpback whales at Georges Bank during the 2006 Gulf of Maine experiment.

**Table 5:** Temporal and spectral characteristics of humpback whale non-song calls.

Non-song calls	Characteristics	Mean	Standard deviation	Minimum	Maximum
“Meows”	Overall call duration (s)	1.44	0.59	0.41	3.60
	Minimum frequency (Hz)	367	45	255	474
	Maximum frequency (Hz)	537	48	410	699
	Repetition interval (s)	31	8	3	50
Series of “Meows”	Overall series duration (s)	300	240	120	840
	Repetition interval (s)	510	288	270	1230
	Number of “Meows”	10	11	2	61
“Bow-shaped” calls	Overall call duration (s)	2.36	0.92	0.69	4.38
	Minimum frequency (Hz)	367	29	269	450
	Maximum frequency (Hz)	511	39	440	600
	Repetition interval (s)	58	2	55	62
“Feeding cries”	Overall call duration (s)	3.18	1.59	1.65	8.10
	Minimum frequency (Hz)	363	23	293	395
	Maximum frequency (Hz)	540	23	492	585
	Repetition interval (s)	692	464	78	1638

These calls include “meows” and “bow-shaped” calls, both of which are primarily uttered in series at night, and “feeding cries”, which only occur at night but far less frequently than “meows” and “bow-shaped” calls. We find that roughly 73% of humpback whale non-song calls are “meows”, roughly 22% are “bow-shaped” calls, and roughly 5% are “feeding cries”.

The diurnal humpback whale call rate (calls/min) time series of Figure 4(A) is obtained by averaging daily humpback whale call rate time series over the entire experiment. The daily humpback whale call rate time series is quantified in 15 minute bins over a diurnal cycle. We define a time period that (1) contains at least two song themes with (2) a gap of silence not exceeding 10 minutes between the adjacent song themes as the occurrence session of humpback whale songs. Similarly, a series of “meows” (Figure 7) or “bow-shaped” calls, and individually uttered non-song calls (Figure 6) constitute the occurrence sessions of repetitive non-song calls and random non-song calls, respectively. A time period longer than 10 minutes containing no calls is defined as the occurrence session of “No calls detected”, and is mutually exclusive with the occurrence sessions of the other three categories. The percentage of time with songs, repetitive non-song calls and random non-song calls, as shown in Figure 5, are quantified using these defined occurrence sessions. The total percentage, the sum of all four categories, may exceed 100% because different types of humpback whale calls may occur simultaneously in overlapping time windows. The number of whales singing at any given time within their detection ranges is found to be consistent with past observations [10], [67], [93], [94], [101], [116]–[118].

### 3.3 Passive position estimation of vocalizing humpback whales with a towed horizontal receiver line-array

To determine the horizontal location of a vocalizing humpback whale, both bearing and range need to be estimated. With our densely sampled, large-aperture horizontal receiver array, bearings of vocalizing humpback whales are determined by time-domain beamforming. Synthetic aperture tracking [29] and the array invariant method [30] are applied to determine the range of vocalizing humpback whales from the horizontal receiver array center. The principle of the synthetic aperture tracking technique [29] is to form a synthetic array by combining a series of spatially separated finite apertures of a single towed horizontal line-array. The array invariant method [30] provides instantaneous source range estimation by exploiting the multi-modal arrival structure of guided wave propagation at the horizontal receiver array in a dispersive ocean waveguide. Position estimation error, or the root mean squared (RMS) distance between the actual and estimated location, is a combination of range and bearing errors. Range estimation error, expressed as the percentage of the range from the source location to the horizontal receiver array center, for the synthetic aperture tracking technique is roughly 2% at array broadside and gradually increases to 10% at 65° from broadside and 25% at 90° from broadside, i.e. near or at endfire [29]. Range estimation error for the array invariant method is roughly 4–8% [29] over all azimuthal directions. Bearing estimation error of the time domain beamformer is roughly 0.5° at broadside and gradually increases to 6.0° at endfire [29]. These errors are determined at the same experimental site and time period as the whale position estimates presented here, from thousands of controlled source signals transmitted by the same source array used to locate the herring shoals presented here [18] and are based on absolute Global Positioning System (GPS) ground truth measurements of the source array's position, which are accurate to within 3–10 meters [119]. More than 90% of vocalizing whales are found to be located 0–65° from the broadside direction of the horizontal receiver array. Position estimation error is then less than 2 km for most of the vocalizing whales localized in Figure 13 since they are found within roughly 40 km of the horizontal receiver array center. This error is over an order of magnitude smaller than the spatial scales of the whale concentrations shown in Figure 13, and consequently has negligible influence on the analyses and results. The measured source locations for all calls are used to generate the whale call rate density maps shown in Figures 1–3 and 13. The source location of each call is characterized by a 2D Gaussian probability density function with mean equal to the measured mean position from synthetic aperture tracking or the array invariant method and standard deviations determined by the measured range and bearing standard deviations. The range standard deviation is 2% for sources located at and near array broadside and increases to 25% for sources located at and near array endfire, based on the range errors of both synthetic aperture tracking and the array invariant method [29]. The bearing standard deviation is 0.5° for sources located at or near array broadside and increases to 6.0° for sources located at or near array endfire [29]. The whale call rate density map is determined by superposition of the 2D spatial probability densities for the source location of each call, normalized by the total measurement time. Left-right ambiguity in determining the bearing of a sequence of source signals in this paper is resolved by changing the array's heading during the reception of the sequence of source transmissions, following the standard method for resolving left-right ambiguity in source bearing for line array measurements in the ocean [16], [29], [120]–[123]. For a far-field point source in free space, bearing ambiguity in line array measurements exists in a conical surface about the array's axis with cone angle equal to the bearing of the source with respect to the array's axis, because the phase speed on the array is identical for far-field sources on this cone at any given frequency. When ambiguity is restricted to source locations in the ocean, only two ambiguous bearings remain, left and right in the horizontal plane about the array's axis, for ranges large compared to the water depth of the source and receiver, as is the case in this paper. To resolve this ambiguity, array heading is varied by an amount 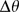 with respect to an absolute coordinate system during the sequence of source transmissions. The true location of the source in absolute coordinates is independent of the array heading, but the bearing of the virtual image source has a component that moves by 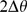 with the array heading. This is analogous to the case where a mirror is rotated by 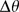, and the true source remains at an absolute position independent of the mirror's orientation but its virtual image in the mirror rotates by an apparent 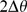 with the mirror's rotation to maintain a specular angle with respect to the mirror's plane and satisfy Snell's Law [21], [124]. The criterion used here to distinguish the virtual image bearing from the true source bearing is that established by Rayleigh [26], [124], [125], where ambiguity is robustly resolved by moving the array heading by an angular amount 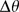 such that the change in virtual bearing 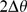 exceeds the array's angular resolution scale (the array beamwidth, Section 3.1) in the direction of the detected source. This Rayleigh resolved change in bearing of the virtual source of 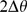 with the array's heading change of 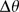 is used to identify the virtual source and distinguish it from the true source, which has an absolute bearing independent of 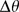. This procedure for ambiguity resolution with the Rayleigh criterion has been applied to all sequences of source transmissions used for source localization in this paper.

### 3.4 Risch et al. statistical test

To evaluate its bias and quantify the impact of this bias, the Risch et al. statistical test of Ref. [34] is applied to Stellwagen Bank humpback whale song occurrence data reported in Refs. [34], [35], since the bias of this test has not been previously investigated, and the implications of a bias have not been previously analyzed or discussed for this test.

The Risch et al. statistical test [34] applies the Tukey method [126] for simultaneous pairwise multiple comparison with the quasi-Poisson generalized linear model (GLM) and log link in the statistical programming language ‘R’ [34], [127], [128] to humpback whale song occurrence over non-overlapping 11-day periods within a 33-day period across years, and tests the resulting pairwise comparisons following the statements of Table 6. The input to the statistical test of Ref. [34] is daily humpback whale song occurrence time series data over each 11-day period. Each pairwise comparison between the mean song occurrence in the 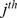 11-day period of the 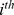 33-day period in the 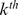 year and that in the 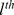 11-day period of the 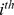 33-day period in the 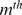 year is assigned a value of 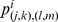. The value of 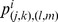 is the probability that the absolute value of the Tukey test statistic [126] is greater than the observed value of the test statistic, conditioned on the null hypothesis, i.e. all mean humpback whale song occurrences over 11-day periods are the same, and is denoted by the variable *P* in Risch et al. [34]. If 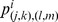 is less than a threshold 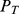 set by the user, then the means are classified by the user to be significantly different, otherwise they are classified by the user to be not significantly different.

**Table 6:** Risch et al. statistical test statements [34].

	Risch et al. Statement	Algorithmic representation
1	“While ‘before’ and ‘after’ periods differed significantly within the years 2008 and 2009 ( 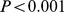 ), with more song recorded in the later period in both years, this increase was not significant in 2006 ( 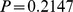 ).”	If 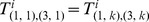 for all 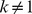 , then 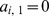 , otherwise 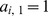 .
2	“In 2006, the ‘during’ period was significantly different from the period ‘after’ ( 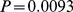 ), with more song recorded later. The 2006 ‘during’ period was not detectably different from the period ‘before’ ( 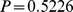 ).”	If 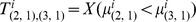 or 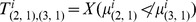 AND 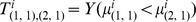 or 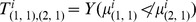 , then 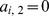 , otherwise 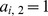 .
3	“When comparing the ‘during’ period across years, 2006 differed significantly from 2009 ( 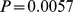 ). The same time period did not differ significantly between 2006 and 2008 ( 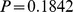 ), or between 2008 and 2009 ( 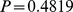 ).”	If 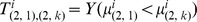 or 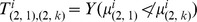 for all 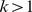 , then 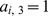 , otherwise 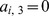 .
4	“Yet, overall there was considerably less song recorded in the 11 ‘during’ days in 2006 compared to both 2008 and 2009.”	If 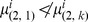 for all 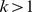 , then 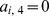 , otherwise 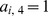 .

Suppose there are daily humpback whale song occurrence time series over *M* years, and for each year there are *N* 33-day periods. Let 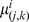 be the mean humpback whale song occurrence over the 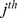 11-day period of the 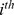 33-day period in the 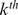 year, where 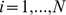, 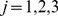, and 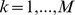. Let 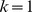 be the test year and let 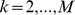 be the control years.

For a given 33-day period over 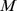 years, there are 
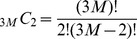
 pairs of 11-day periods. Comparing the 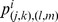 with 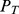 for each of the 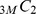 pairs, outcome 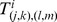 is assigned for the comparison between the mean song occurrence pair 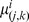 and 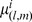. The possible outcomes 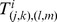 are (1) 
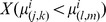
, which is defined as: 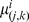 and 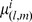 are not significantly different and 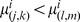; (2) 
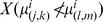
, which is defined as: 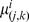 and 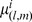 are not significantly different and 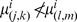; (3) 
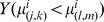
, which is defined as: 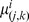 and 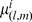 are significantly different and 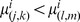; and (4) 
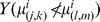
, which is defined as: 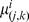 and 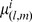 are significantly different and 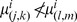, as given in Table 7.

**Table 7:** Possible outcomes of each pairwise comparison between the mean humpback whale song occurrence in the 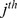 11-day period of the 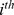 33-day period in the 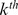 year and that in the 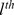 11-day period of the 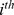 33-day period in the 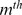 year in the Risch et al. statistical test [34].

Outcome 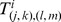	Description
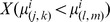	Means are not significantly different and 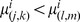
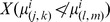	Means are not significantly different and 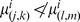
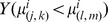	Means are significantly different and 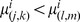
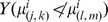	Means are significantly different and 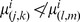

The rate of false positive findings that whales respond to sonar when no sonar is present is
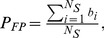
(1)where



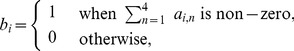
(2)

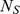
 is the number of 33-day periods when no sonars are present, the 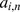 are defined in Table 6, and each 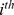 33-day period, for 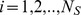, has no sonar present.

#### 3.4.1 False positive rate and statistical bias of the Risch et al. statistical test

When the Risch et al. statistical test [34], as described mathematically in Section 3.4 and Table 6, is applied to the three 33-day humpback whale song occurrence time series data reported in Risch et al. [34], with 11-day time series indices 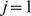 for the “before” period from September 15 to September 25, 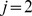 for the “during” period from September 26 to October 6, and 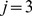 for the “after” period from October 7 to October 17, and indices 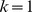 for year 2006, 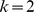 for year 2008 and 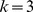 for year 2009, we obtain the same 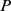 values and results reported in the ‘Risch et al. Statement’ column of Table 6. Specifically, daily humpback whale song occurrence time series denoted by 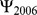 for year 2006, 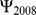 for year 2008, and 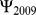 for year 2009, from song occurrence data reported in Risch et al. [34] over the 33-day period from September 15 to October 17, are input to the Tukey tests of the statistical programming language ‘R’, as described in Section 3.4. Since there is only one 33-day period from September 15 to October 17, 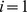. This 33-day period consists of the three consecutive non-overlapping 11-day periods with indices 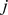 or 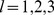 and year indices 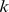 or 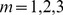 for pairwise comparisons between periods within and across years. A value of 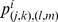, the 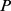 value, and a corresponding 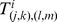 outcome are determined for each pairwise comparison between the mean song occurrence in the 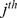 11-day period of the 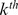 year and that in the 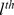 11-day period of the 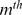 year from the Tukey tests, as described in Section 3.4.

We apply the Risch et al. statistical test [34] to the two-year humpback whale song occurrence daily time series data reported in Vu et al. [35] with the same statistical test settings used to obtain the *P* values and results reported in the ‘Risch et al. Statement’ column of Table 6. The Vu et al. [35] daily humpback whale song occurrence time series (Figure 3 of Ref. [35]) over the 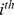 33-day period, denoted by 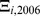 for year 2006 and 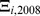 for year 2008, are input to the Tukey tests of the statistical programming language ‘R’, as described in Section 3.4. For the 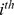 33-day period, consisting of three consecutive non-overlapping 11-day periods with indices 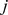 or 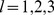, and year indices 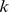 or 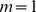 for the test year 2006 and 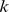 or 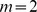 for the control year 2008, a value of 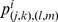, the 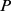 value, and a corresponding 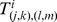 outcome are determined for each pairwise comparison between the mean song occurrence in the 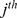 11-day period of the 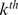 year and that in the 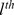 11-day period of the 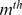 year from the Tukey tests, as described in Section 3.4. From the outcomes 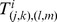, the corresponding 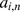 are determined based on Table 6. This is repeated for all continuous 33-day periods, where the 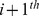 33-day period begins 1-day after the 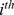 33-day period. Only 33-day periods that have 11-day periods with reported whale song occurrence are included. If data is missing in any day from a 33-day period, then that 33-day period is excluded from both years. False positive rates are then determined from 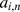 via Equations (1) and (2). The Risch et al. statistical test [34] false-positively finds whales react to sonar in (a) 100% of the 35 continuous 33-day periods before the “during” period (Table 1) when no sonar is present; and (b) 98% of the 50 continuous 33-day periods excluding the “during” period (Table 1) when no sonar is present. No valid or meaningful conclusions can be drawn from such an overwhelmingly biased statistical test. This specific application of the Risch et al. statistical test [34] has not been previously reported.

When the Risch et al. statistical test [34] is applied to the same humpback whale song occurrence data, 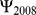 and 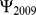, reported in Risch et al. [34] over the 33-day period between September 15 and October 17, with 11-day time series indices 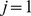 for the “before” period, 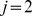 for the “during” period, and 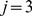 for the “after” period, and year indices 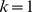 for the test year 2008 and 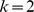 for the control year 2009, as well as with year indices 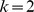 for the control year 2008 and 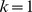 for the test year 2009, the test false-positively finds that whales react to sonar 100% of the time when no sonar is present, indicating self-contradictions in the Risch et al. [34] approach, as shown in Table 2, which make their analysis and conclusions invalid. This specific application of the Risch et al. statistical test [34] has also not been previously reported.

### 3.5 Model for detectable humpback whale song occurrence

Detectable humpback whale song occurrence for a coherent sensor array can be quantified in terms of local wind-speed-dependent ambient noise for a given spatial distribution of vocalizing humpback whales. The humpback whale song occurrence depends on the presence of at least one singing humpback whale inside the mean wind-dependent detection range of the sensor array. The percentage of time in a day over which a humpback whale is within the mean detection area and is singing corresponds to the measured daily humpback whale song occurrence rate.

The detection range [20], [23], [25], [39], [71], 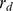, is defined as the range from the center of the array at which signals, in this case humpback whale songs, can no longer be detected above the ambient noise, and is the solution of the sonar equation [20]–[24],
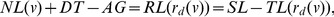
(3)where 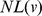 is the wind-speed-dependent ambient noise level, 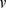 is the wind speed, 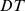 is the detection threshold, 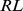 is the received sound pressure level due to a humpback whale song source level 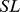 undergoing a transmission loss of 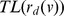 at range 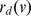 for some given source and receiver depths, and 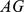 is the array gain equal to 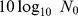 for a horizontal array, where 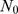 is the number of coherent sensors spaced at half wavelength [20]–[24]. The capability of sensor arrays with high array gain such as ours to detect sources orders of magnitude more distant in range than a single sensor is standard, well established and well documented in many textbooks [20]–[24], [27]. The array gain of our coherent horizontal OAWRS receiver array is 18 dB, which enables detection of whale vocalizations in an ocean acoustic waveguide [20], [22], [24], [27] up to either two orders of magnitude lower in SNR or two orders of magnitude more distant in range than a single hydrophone [20]–[24], [27], which has zero array gain [20]–[24], [27], by direct inspection of Equation (3). We set the detection threshold, DT, such that the sum of signal and noise is detectable at least 5.6 dB [129]–[132] above the noise. The ambient noise and the received signal are filtered to the frequency band of the source. Further, the wind-speed-dependent ambient noise level is modeled as
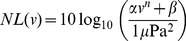
(4)where 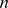 is the power law coefficient of wind-speed-dependent ambient noise, 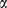 is the waveguide propagation factor [133] and 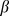 corresponds to the constant baseline sound pressure squared in the frequency band of the source. The coefficients 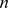, 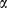 and 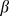 are empirically obtained by minimizing the root mean square error between the measured and the modeled ambient noise level as a function of measured wind speed during the OAWRS experiment in the Gulf of Maine [18]. We find 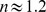 in the frequency range of the observed humpback song units, which is consistent with past ambient noise measurements in high shipping traffic regions [134]–[137]. (A value of 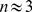 would have been consistent with wind-dependent ambient noise with no significant shipping component [138]–[140] but a value of 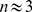 was not obtained.) The noise levels obtained from Equation (4) in Stellwagen Bank are consistent with those reported in Risch et al. [34].

A standard parabolic equation model of the US Navy and the scientific community, Range-dependent Acoustic Model (RAM) [22], [141]–[144], that takes into account range-dependent environmental parameters is used to calculate the transmission loss 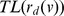 from the whale location to the sensor in a highly range-dependent continental-shelf environment in the Gulf of Maine including Stellwagen Bank. The model uses experimentally measured sound speed profiles acquired during the OAWRS 2006 experiment [19] and standard bathymetry data for the Gulf of Maine [145]. Expected transmission loss [146] is determined along any given propagation path from source to receiver by Monte-Carlo simulation over range-dependent bathymetry [145] and range-dependent sound speed structures measured from oceanographic data [19], [55], [147], [148]. An estimate of detection range 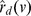 for a given humpback whale song unit source level can be obtained from Equation (3) by a minimum mean squared error method. Higher transmission loss occurs in shallower waters due to more intense and pervasive bottom interaction [20]–[24]. Transmission loss in deeper waters is typically significantly lower due to upward refraction [20], [22] which leads to far less intense and pervasive bottom interaction, as is the case in the deeper waters surrounding Georges Bank [20]–[24]. Highly directional transmission loss may then occur when there are large depth variations about a receiver. Indeed, this effect makes the detection range of whales in directions to the North of our receiver and Georges bank much greater than in directions to its South where the relatively shallow waters of Georges Bank are found (Figure 13). The fact that we localized the sources of many whale calls at great distances along shallow water propagation paths on Georges Bank in directions where transmission loss was greater and found negligibly small vocalization rates much closer to the receiver in the deeper waters north of Georges Bank where transmission loss was much less greatly emphasizes the finding that the vocalization rates originating from north of Georges Bank were negligibly small. This indeed is expected based on general behavioral principles [33] since the whales' dominant prey was on Georges Bank, where the majority of whale vocalizations originated (Figures 1–3), and not in the deeper waters to the North, as we note in Section 2.1. This is also consistent with the historical distribution of humpback whales in the Gulf of Maine during the fall season [45]. The ranges and propagation paths from deep to shallow waters between our receiver array and Stellwagen Bank are very similar to those between our receiver array and the distant whale call sources localized along Georges Bank (Figure 13). The corresponding transmission losses have negligible differences. The fact that we localized the sources of many whale calls on Georges Bank but found negligibly small vocalization rates originating from Stellwagen Bank in the “before” or “during” periods, then emphasizes the fact that vocalization rates originating from Stellwagen Bank were negligibly small in these periods. As noted in Sections 2.1 and 2.2, this is consistent with the well documented findings that humpback whales migrate away from Stellwagen Bank where herring stocks have collapsed to feed at other locations that support large herring aggregations such as Georges Bank [33]. Our transmission loss calculations with the standard RAM parabolic equation model have been extensively and successfully calibrated and verified with (1) thousands of one-way transmission loss measurements made during the same 2006 Gulf of Maine experiment discussed here at the same time and at the same location [19], [149]; (2) thousands of two-way transmission loss measurements made from herring shoal returns and verified by conventional fish finding sonar and ground truth trawl surveys during the same 2006 Gulf of Maine experiment discussed here at the same time and at the same location [18], [19],[150]; (3) roughly one hundred two-way transmission loss measurements made from calibrated targets with known scattering properties during the same 2006 Gulf of Maine experiment discussed here at the same time and at the same location [151]; and (4) thousands of one-way transmission loss measurements made during a past OAWRS experiment conducted in a similar continental shelf environment [147].

**Figure 17:**
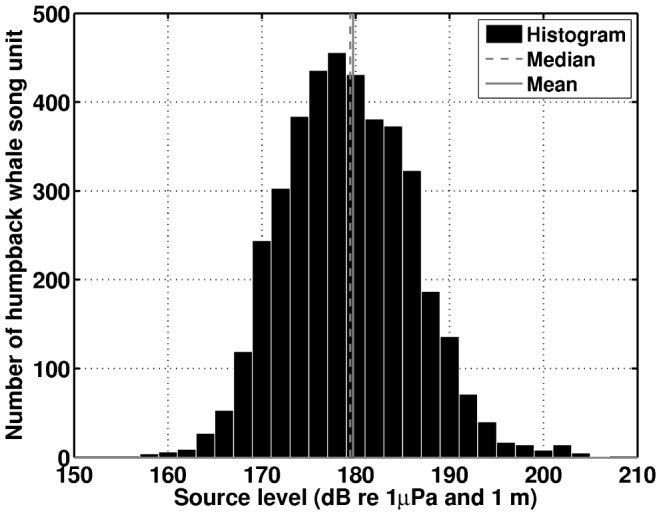
Histogram of the measured humpback whale song unit source levels. The humpback whale song unit source levels measured from more than 4,000 recorded song units during the same 2006 Gulf of Maine experiment discussed here at the same time and at the same location approximately follow a Gaussian distribution and are in the range 155 to 205 dB re 1 *µ*Pa and 1 m with a mean of 179.8 dB re 1 *µ*Pa and 1 m and a median of 179.4 dB re 1 *µ*Pa and 1 m, which are within 0.6 dB of the median of all published humpback whale song unit source levels of 180 dB re 1 *µ*Pa and 1 m [93], [101], [102], [152]–[154]. The solid and dashed gray lines represent the mean and the median of the measured humpback song unit source levels, respectively.

We find that the humpback whale song source levels measured from more than 4,000 song units recorded during the same 2006 Gulf of Maine experiment discussed here at the same time and at the same location approximately follow a Gaussian distribution and are in the range 155 to 205 dB re 1 µPa and 1 m (Figure 17) with a mean of 179.8 dB re 1 µPa and 1 m and a median of 179.4 dB re 1 µPa and 1 m. The high array gain [20]–[25] of our densely sampled, large aperture coherent OAWRS horizontal receiver array used here enables detection of whale songs two orders of magnitude lower in SNR than a single hydrophone, which has no array gain. Our measurements of humpback whale song source levels then have a high dynamic range and span the wide range of published source levels [9], [93], [101], [102], [152], [153], except for those in Ref. [154], which appear to be anomalously low compared to the rest of the literature as has been previously noted in Ref. [9]. The mean and median of our measured source levels match very well (within 0.6 dB) with the median of all published humpback whale song unit source levels of 180 dB re 1 µPa and 1 m [93], [101], [102], [152]–[154]. Our song unit source levels are determined given our estimated whale positions and waveguide propagation modeling. Results in Figures 14 and 15 are computed using our measured whale positions and the median of all published humpback song source levels of 180 dB re 1 µPa and 1 m [93], [101], [102], [152]–[154], which has negligible difference from our measured median and mean song source levels, for the range of measured humpback singing depths of 2 m to 25 m [152], [155]. Results in Figures 14 and 15 are insensitive to variations in whale position variations within the errors we report for our measured whale positions in Section 3.3, and so are insensitive to the whale position errors of our measurement system. Insensitivity here means the measured to modeled song occurrence match is within ±18% as in Figure 15.

The total humpback whale song occurrence in a day detectable by a sensor in varying wind speeds is
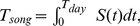
(5)where 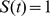 when 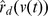 is greater than or equal to the minimum of 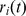 over all 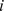, and 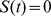 when 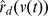 is less than the minimum of 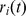 over all 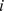, where 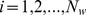, 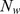 is the total number of singing whales, 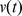 is the measured wind speed, 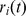 is the range of the 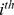 singing humpback whale from the sensor at time 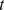, and 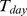 is the full diurnal time period of 24 hours. The detectable humpback whale song occurrence rate is then 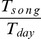.

### 3.6 Autocorrelation of annual humpback whale song occurrence time series in 2006 and 2008

**Figure 18:**
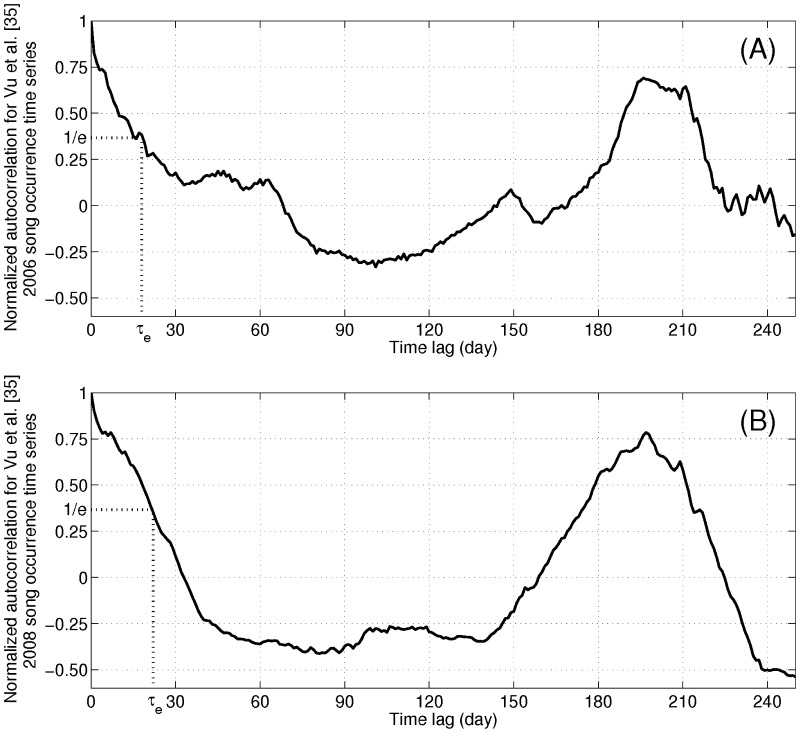
Autocorrelation of Vu et al. [35] humpback whale song occurrence time series in 2006 and 2008. The e-folding time scale 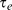 of the Vu et al. [35] annual humpback whale song occurrence time series is (A) 18 days for 2006 and (B) 21 days for 2008. The roughly 20-day coherence time scale shows that the humpback song occurrence gradually changes over periods longer than the 11-day periods analyzed in Risch et al. [34]. It is noteworthy that (1) the humpback song occurrence dropped to zero in the “before” period, and (2) only after a time period consistent with the measured coherence time scale of song occurrence, within which temporal processes are correlated, did song occurrence begin to increase in the “during” period (Figure 10). The Risch et al. [34] analysis then violates temporal causality because the correlated processes that caused the reduction in humpback song occurrence started days before the OAWRS survey transmissions began, yet the analysis and conclusions of Risch et al. [34] offer no other explanation than these survey transmissions for the reduction. Both time series show high correlation at a time lag of roughly seven months due to increases in song occurrence during the spring and fall seasons (Figure 10), separated by roughly seven months.

We calculated the normalized autocorrelation function [156] of the Vu et al. [35] 2006 and 2008 annual humpback whale song occurrence time series. The autocorrelation function at zero time lag, where perfect temporal correlation exists, is one. The time lag at which the autocorrelation function falls to 1/*e* is the e-folding time scale defining the width of the correlation peak, or coherence time scale, within which processes are conventionally taken to be correlated [156], [157]. The e-folding time scale of the Vu et al. [35] annual humpback whale song occurrence time series is 18 days for 2006 and 21 days for 2008 (Figure 18). The roughly 20-day coherence time scale shows that the humpback song occurrence gradually changes over periods longer than the 11-day periods analyzed in Risch et al. [34]. This time is consistent with the smooth and gradual transition in Figure 12 of the correlation coefficient of 11-day periods across years from negative values in the “before” period to some of the highest positive correlations obtained between years in the “during” period, which contradicts the results of the Risch et al. [34] study and is consistent with a violation of temporal causality in the Risch et al. [34] study. It is noteworthy that (1) the humpback song occurrence dropped to zero in the “before” period, and (2) only after a time period consistent with the measured coherence time scale of song occurrence, within which temporal processes are correlated, did song occurrence begin to increase in the “during” period (Figure 10). The Risch et al. [34] analysis then also violates temporal causality because the correlated processes that caused the reduction in humpback song occurrence started days before the OAWRS survey transmissions began, yet the analysis and conclusions of Risch et al. [34] offer no other explanation than these OAWRS survey transmissions for the reduction, when only other causes are causally possible. Indeed as we have shown in Section 2.2 non-sonar causes regularly lead to such changes in song occurrence, and as we have shown in Section 3.5 standard detection range variations from measured wind speed dependent noise variations at Stellwagen and measured humpback whale song sources near Georges Bank completely account for the changes reported in Risch et al. [34].
